# Freeze-Thaw Pretreatment Can Improve Efficiency of Bacterial DNA Extraction From Meconium

**DOI:** 10.3389/fmicb.2021.753688

**Published:** 2021-12-09

**Authors:** Yuntian Xin, Jingxian Xie, Bingru Nan, Chen Tang, Yunshan Xiao, Quanfeng Wu, Yi Lin, Xueqin Zhang, Heqing Shen

**Affiliations:** ^1^Key Laboratory of Urban Environment and Health, Institute of Urban Environment, Chinese Academy of Sciences, Xiamen, China; ^2^Department of Obstetrics, Women and Children’s Hospital, School of Medicine, Xiamen University, Xiamen, China; ^3^State Key Laboratory of Molecular Vaccinology and Molecular Diagnostics, School of Public Health, Xiamen University, Xiamen, China; ^4^University of Chinese Academy of Sciences, Beijing, China

**Keywords:** meconium, DNA extraction, 16S rDNA, gene sequencing, gut microbiome

## Abstract

Although the presence of live microbes *in utero* remains under debate, newborn gastrointestinal bacteria are undoubtedly important to infant health. Measuring bacteria in meconium is an ideal strategy to understand this issue; however, the low efficiency of bacterial DNA extraction from meconium has limited its utilization. This study aims to improve the efficiency of bacterial DNA extraction from meconium, which generally has low levels of microflora but high levels of PCR inhibitors in the viscous matrix. The research was approved by the ethical committee of the Xiamen Maternity and Child Health Care Hospital, Xiamen, China. All the mothers delivered naturally, and their newborns were healthy. Meconium samples passed by the newborns within 24 h were collected. Each sample was scraped off of a sterile diaper, transferred to a 5-ml sterile tube, and stored at −80°C. For the assay, a freeze-thawing sample preparation protocol was designed, in which a meconium-InhibitEX buffer mixture was intentionally frozen 1–3 times at −20°C, −80°C, and (or) in liquid nitrogen. Then, DNA was extracted using a commercial kit and sequenced by 16S rDNA to verify the enhanced bacterial DNA extraction efficiency. Ultimately, we observed the following: (1) About 30 mg lyophilized meconium was the optimal amount for DNA extraction. (2) Freezing treatment for 6 h improved DNA extraction at −20°C. (3) DNA extraction efficiency was significantly higher with the immediate thaw strategy than with gradient thawing at −20°C, −80°C, and in liquid nitrogen. (4) Among the conditions of −20°C, −80°C, and liquid nitrogen, −20°C was the best freezing condition for both improving DNA extraction efficiency and preserving microbial species diversity in meconium, while liquid nitrogen was the worst condition. (5) Three freeze-thaw cycles could markedly enhance DNA extraction efficiency and preserve the species diversity of meconium microflora. We developed a feasible freeze-thaw pretreatment protocol to improve the extraction of microbial DNA from meconium, which may be beneficial for newborn bacterial colonization studies.

## Introduction

The human intestinal microflora is a highly diversified ecosystem composed of trillions of gastrointestinal bacteria with counts approximately 10 times the number of human cells (Bäckhed et al., 2005; Biagi et al., 2017). These microorganisms form the “human biome” by coexisting with the host (Sundman et al., 2017). Many of their signaling molecules and/or metabolites play important roles in innate and adaptive immune responses (O'Callaghan et al., 2016), maintenance of the host immune system (Deriu et al., 2016), protection of the host from pathogen invasion, and synthesis of essential vitamins and nutrients (Buffie et al., 2015). The intestinal microflora has the function of transforming nutrients into endocrine signals, which can not only affect metabolism inside the intestine (Eckburg et al., 2005) but also affect the gut nervous system (Hooper and Gordon, 2001). Alterations in the intestinal flora can cause obesity, colorectal cancer (CRC), diabetes, cardiovascular disease, liver disease, and other diseases (Loscalzo, 2013; Qian et al., 2015; Aron-Wisnewsky and Clément, 2016; Forsythe et al., 2016; Gensollen et al., 2016).

The presence of bacteria has been considered to lead to systemic inflammatory reactions and multiple organ damage, which pose a threat to the growth and development of the foetus and can even lead to abortion, premature birth, and stillbirth. During normal pregnancy, foetal growth and development *in utero* have been widely accepted to occur in a sterile environment. In 2008, *Enterococcus* and *Staphylococcus* were mainly identified in 21 healthy newborns by investigating their meconium (Jiménez et al., 2008); this work ignited an ongoing debate regarding whether the *in utero* environment is sterile. In 2010 (Mshvildadze et al., 2010), microbial DNA in meconium was detected, and meconium was confirmed to be rich in bacteria in 2014 (Guo, 2014), implying that infants may have started to establish their intestinal flora before birth. In 2015 (Hansen et al., 2015), evidence demonstrated a low number of bacteria in first-pass meconium from healthy, vaginally delivered, breastfeeding infants. Research on 151 vaginally born or Caesarean-section-born healthy babies in 2016 showed that several bacterial branches may already exist in the intestines of term infants (Nagpal et al., 2016). Bacterial 16S rRNA genes were characterized in first-pass meconium samples from 218 newborns in 2018 at the same hospital (Tapiainen et al., 2018), which revealed the general profiles of infants’ fecal microorganisms to some extent. Studies in 2019 further showed that all meconium and most amniotic fluid samples contain bacterial DNA (Stinson et al., 2019; Willis et al., 2019). In summary, human meconium contains complex microbial communities, and these bacteria may have and may continue to affect the development of the foetal immune system and host-microbe interactions.

Meconium is a viscid, odorless, greenish-black material that is quite different from watery and light-yellow newborn stools (Bearer, 2003). Usually, meconium begins to form *in utero* at approximately the 13th week of gestation and accumulates in the foetus until birth. Meconium is passed naturally by full-term neonates within 48 h after birth (Lisowska-Myjak and Pachecka, 2006, 2007). Meconium is a multicomponent mixture that can include bile pigments, water-soluble substances, such as proteins, and chloroform-soluble substances containing unsaturated lipids, fatty acids, and cholesterol (Pallem et al., 2010). These components originate from the foetus swallowing amniotic fluid containing shed epithelial cells and intestinal secretions (Harries, 1978; Sun et al., 1993). To some extent, the components in meconium can reflect the developmental environment of the foetus in the womb, and increasing attention has been devoted to investigating the matrix, including bacteria. However, studying meconium is challenging. One of the main problems is that the existence of microbial communities in the uterine environment is still controversial. Such debate was mainly due to the following problems: (i) The prokaryotic DNA content is approximately 1 % in 1-year-old infant stool samples (Wampach et al., 2017), and only a small proportion of extracted DNA can amplify the prokaryote bands (Hansen et al., 2015). However, current researches used DNA-based approaches with an insufficient detection limit to study “low-biomass” microbial communities; (ii) molecular assessments lacked appropriate controls for contamination; and (iii) failed to provide evidence of bacterial viability. Additionally, previous study stated if microbes were detected, bacteria in the first stool of the newborn could also be contributed to the result of postnatal colonization (Perez-Muñoz et al., 2017).

Scientists have suggested that the reason for the low efficiency of DNA extraction in meconium derives from the presence of many PCR inhibitors in the matrix. These inhibitors can be bile acids and salts (Al-Soud et al., 2005), glycolipids (Karlsson and Larson, 1978), and urea (Schrader et al., 2012), which are difficult to remove during the DNA extraction process and can cause difficultly for subsequent experiments. In addition, the absolute content of microorganisms in meconium may also lead to a low DNA detection rate. Low levels of bacterial DNA are usually caused by high concentrations of PCR inhibitors in meconium (Villanueva et al., 2000; Hansen et al., 2015), and the amplification rate of extracted DNA in meconium samples is commonly very low (10–52.9%; Ardissone et al., 2014; Hansen et al., 2015). Establishing a meconium-specific sampling extraction protocol is important for investigating the microbiome in the newborn gut (Stinson et al., 2018).

In the present study, a commercial QIAamp® Fast DNA Stool Mini Kit (cat. no. 51604) was used to develop the extraction protocol for DNA in meconium. We found that freeze-thaw treatment of meconium in lysis buffer can increase the DNA yield. As a result, we established a freeze-thaw procedure and verified the factors affecting the efficiency of DNA extraction.

## Materials and Methods

### Sample Collection

The research was approved by the ethics committee of the Xiamen Maternity and Child Health Care Hospital (XMCH), Xiamen, China. The newborns were delivered at the Women and Children’s Hospital, School of Medicine, Xiamen University, from June 1st, 2017, to August 15th, 2017. When written informed consent was provided, the participants were recruited according to the following criteria: (1) gestational age between 37 and 42 weeks; (2) fulfilment of regular prenatal visits and complete clinical data; and (3) full-term deliveries. Pregnant women with the following complications were excluded: (1) infectious diseases caused by bacteria, viruses, or parasites; (2) inflammatory diseases (e.g., ankylosing spondylitis); (3) metabolic diseases such as diabetes mellitus; (4) pregnancy-associated illnesses including preeclampsia and gestational hypertension; (5) reproductive system disorders (e.g., an ovarian cyst); (6) abnormal pregnancy state (e.g., preterm birth); (7) genetic diseases such as thalassemia; and (8) tumors including pituitary adenomas and uterine fibroids. All mothers delivered naturally, and their newborns were healthy. Meconium was passed by the newborns within 24 h, and samples were collected immediately from sterile baby diapers. Samples were scraped off from baby diapers with sterilized metal scalpel handles and transferred to 5.0-ml sterilized tubes for subsequent analysis. Each meconium sample was divided into two fractions: one part was numbered and directly stored at −80°C, the other part was lyophilized under vacuum conditions, and the freeze-dried samples were numbered and stored at −80°C.

### DNA Extractions Methods

In this study, protocols for extracting DNA in meconium were explored using the basic protocol (blue blocks in Supplementary Figure S1) of the commercial QIAamp® Fast DNA Stool Mini Kit (cat. no. 51604). Because of the poor dispersion of meconium in the InhibitEX buffer, each sample was first mixed with 1 ml InhibitEX buffer and vortexed for 30 min. Then, a mixture of meconium and InhibitEX buffer was obtained and named the meconium lysis cocktail. Extra treatments (red block in Supplementary Figure S1) were applied to the cocktail before the following steps of DNA extraction (these remaining steps were consistent with the instructions offered by the kit manufacturer). The treatments were designed to improve DNA excretion from the meconium matrix, and factors that may affect excretion were assessed. All experimental details for these factors are listed as follows:

1. Status and amount of meconium: DNA was extracted from either fresh or freeze-dried meconium, and the extraction efficiency was assessed to test which conditions are better for fresh meconium (40, 80, 120, 160, and 200 mg) and freeze-dried meconium (10, 20, 30, 40, 50, and 60 mg). These conditions were tested with five duplicate samples for each test.
2. Freezing time for the meconium lysis cocktails: For DNA extraction, the cocktail samples were intentionally frozen for 0, 6, 12, 18, 24, 36, and 48 h at −20 or −80°C. The remaining steps were performed by following the basic steps in the protocol (Supplementary Figure S1). All samples were freeze-dried meconium, and each sample weighed approximately 30 mg.
3. DNA extraction efficiency with immediate and gradient thawing: After vortexing, the meconium lysis cocktails were immediately frozen at −20 or −80°C or in liquid nitrogen for 6 h. Then, different thawing methods were carried out, which involved either immediate thawing or gradient thawing. These tests were designed to assess both freezing temperatures and thawing methods, where the frozen meconium lysis cocktails were thawed immediately or were thawed according to gradient programs (Supplementary Figure S2): ① −20°C-frozen meconium lysis cocktails were transferred to 4°C for 6 h. ② −80°C-frozen meconium lysis cocktails were transferred to 4°C for 6 h. ③ Meconium lysis cocktails frozen at −80°C were transferred to −20°C for 6 h and then transferred to 4°C for 6 h. ④ Liquid nitrogen-frozen meconium lysis cocktails were transferred to 4°C and incubated for 6 h. ⑤ Liquid nitrogen-frozen meconium lysis cocktails were transferred to −80°C for 6 h, then transferred to −20°C for 6 h, and finally transferred to 4°C for 6 h. After the thawing steps, the remaining steps all followed the DNA extraction kit instructions. Each sample was a 30-mg freeze-dried meconium specimen.
4. DNA extraction efficiency with immediate and gradient freezing: The measures of immediate and gradient freezing for the meconium lysis cocktails were tested after vortexing (Supplementary Figure S3). For immediate freezing, the meconium lysis cocktails were frozen immediately for 6 h at −80°C or in liquid nitrogen. For gradient freezing treatments, the cocktails were frozen stepwise until the target temperatures were reached: ① the cocktails were frozen at −20°C for 6 h and then transferred to −80°C for 6 h; ② the cocktails were frozen at −20°C for 6 h and then transferred to liquid nitrogen for 6 h; and ③ the cocktails were frozen at −20°C for 6 h and then transferred to −80°C for 6 h and in liquid nitrogen for 6 h. After the freezing step, the samples were thawed immediately for the remaining steps of DNA extraction. Each sample was a 30-mg freeze-dried meconium specimen.
5. DNA extraction efficiency with freeze-thaw recycling: The meconium lysis cocktails were immediately frozen at −20 and −80°C and in liquid nitrogen for 6 h to verify the efficiency of DNA extraction for duplicate freeze-thaw cycles. For each cycle, the cocktails were immediately thawed at room temperature. The freeze-thaw operation was carried out for one, two, and three cycles (Supplementary Figure S4). Finally, the thawed meconium lysis cocktails were subjected to the remaining steps of DNA extraction. Each sample was a 30-mg freeze-dried meconium specimen.

### Quantitative Real-Time PCR

All DNA samples were amplified by quantitative real-time PCR (qPCR) for the hypervariable V3-V4 region of the 16S rRNA gene to verify the DNA extraction efficiency. The PCR system contained 10 μl Roche LightCycler® 480 SYBR® Green I Master, 1 μl of 10 μM each of the forward (5′-CCTAYGGGRBGCASCAG-3′) and reverse (5′-GGACTACHVGGGTWTCTAAT-3′) primers (final concentration 0.5 μM), 5 μl of template or water (negative template control), and 3 μl of water. All samples, including the reference controls, were run in duplicate. The instrument used in this assay was a Roche LightCycler 480II. The amplification procedure was as follows: predenaturation at 94°C for 10 min; 40 cycles of denaturation at 94°C for 30 s, annealing at 55°C for 30 s, and extension at 72°C for 30 s; melting curves of 94°C for 5 s and 65°C for 1 min; continuous collection of the fluorescent data until 97°C; and cooling. In practice, the DNA amplification efficiency is usually lower than the theoretical value of 100%. To acquire high-quality data, a group of standard curves was set, and the calculated amplification efficiency was approximately 80% in this assay.

### 16S rDNA Sequencing

To further evaluate the effect of freezing treatment on meconium bacterial diversity, in addition to testing the impact of freezing treatment on DNA extraction efficiency, six different meconium matrixes were treated by using seven different DNA extraction methods. The protocols were meconium lysis cocktails frozen at −20 or −80°C or in liquid nitrogen and treated with one-, two-, and three-freeze-thaw cycles. The samples were coded by the method name (frozen methods tandem freeze-thaw cycle number) plus meconium identification. The meconium identifications were coded as A, B, C, D, E, and F; the frozen methods were marked as 20, 80, and N for −20°C, −80°C, and liquid nitrogen, respectively, and the freeze-thaw cycle number was marked as I, II, and III, respectively. For example, A80II represented meconium A that had been frozen at −80°C and underwent two freeze-thaw recycling cycles. All extracted DNA was sequenced by 16S rDNA sequencing technology. 16S rDNA is the DNA sequence corresponding to the rRNA encoded in bacteria that exists in all bacterial genomes. The 16S rDNA in this assay was sequenced on an Illumina HiSeqTM 2500 by Gene Denovo Biotechnology Co., Ltd. (Guangzhou, China). And the raw data microbiome sequencing was uploaded into NCBI database under the accession number of PRJNA759695.

### Data Analysis

All data were processed by SPSS19 and Excel. Student’s *t*-test was used to verify the significance of differences in the intergroup data, and all *p*-values were applied to the two-sided tests unless otherwise specified. Bioinformatic analysis was performed using Omicsmart, which is a dynamic real-time interactive online platform for data analysis.^1^

## Results and Discussion

### Meconium Status and Loading Amount Affect DNA Extraction Efficiency

The recommended sample for the kit was a 200-mg fresh sample. As shown in Supplementary Table S1, the water content of meconium was approximately 60–70% in this assay calculated by the following formulation:


$$H_{2}O\;\left(\%\right)=\left(\mathrm{WW}-\mathrm{DW}\right)/\mathrm{WW}\times 100\%$$

Among them, WW and DW represented the wet and dry weight of meconium, respectively. Therefore, 200 mg fresh meconium and 60 mg freeze-dried meconium were initially applied in the protocol development.

**Figure 1:**
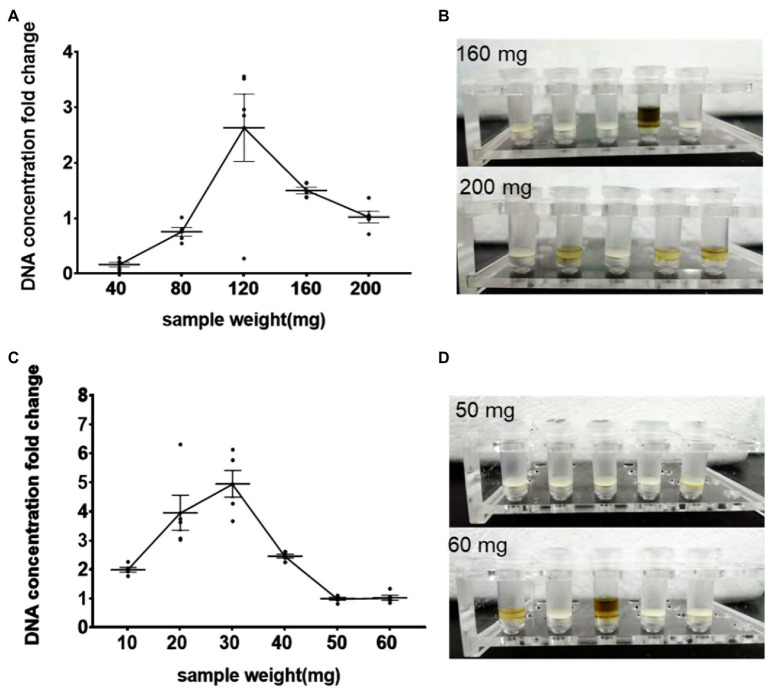
Effects of meconium status and loading amount on DNA extraction efficiency. **(A)** DNA concentration (fold change) vs. fresh meconium loading amount (mg); **(B)** Columns could be blocked by overloaded fresh samples (loading with 160 and 200 mg after centrifugation); **(C)** DNA concentration (fold change) vs. freeze-dried meconium loading amount (mg); **(D)** Columns could be blocked by overloaded freeze-dried samples (loading with 50 mg and 60 mg after centrifugation). The results are presented as the average of five duplicates ± SEM. The corresponding data were shown in Supplementary Table S2.

The relationship between DNA extraction efficiency and sample weight presented an inverted U-shape for both fresh and freeze-dried samples (Figure 1; Supplementary Table S2). The DNA extraction efficiency reached the peak values when fresh meconium weighed 120 mg (Figure 1A) and freeze-dried meconium weighed 30 mg (Figure 1C). The DNA extraction efficiency of the frozen samples was generally higher than that of the fresh samples. In addition, both fresh samples (Figure 1B) and dry samples (Figure 1D) could clog the column filters when they were overloaded as liquid above the adsorption column could not enter the lower side. When comparing the DNA extraction efficiency between fresh and freeze-dried meconium, the overall efficiency for freeze-dried meconium was higher than that for fresh meconium.

The above results showed that the sample status and loading amount were two important factors in DNA extraction. Overloading can cause a lower DNA extraction efficiency than proper loading, possibly because of the following reasons: (1) Samples cannot be fully lysed to release DNA. (2) The fragments of samples blocked the filter column, which affected the subsequent operations. (3) More PCR inhibitors (Villanueva et al., 2000; Hansen et al., 2015) were loaded in the overloaded meconium. Interestingly, freeze-dried meconium showed a better DNA extraction efficiency than fresh meconium, possibly because freeze-dried meconium is homogeneous. Then, 30 mg freeze-dried meconium was chosen in the final protocol.

### Freezing Pretreatment Affects Meconium DNA Extraction Efficiency

Accidentally, we observed that freezing meconium in lysis buffer can increase the DNA yield. Because some physical treatments (van Burik et al., 1998; Salonen et al., 2010) can improve the efficiency of DNA extraction more obviously than chemical treatments (Rohland and Hofreiter, 2007), the observational improvement encouraged the following experimental designs to test the effects of freezing time, freezing temperature, thawing conditions, and freeze-thaw cycles on the DNA extraction efficiency.

#### Freezing Time for Meconium

To determine the appropriate time for meconium freezing, two freezing temperatures of −20 and −80°C were assessed in both fresh and freeze-dried meconium at times ranging from 0 to 48 h. The results showed that the DNA extraction efficiency of the frozen samples was generally higher than that of the control, and the efficiency at −20°C was higher than that at −80°C. Similar time trends for the changed DNA extraction efficiencies were observed for the two freezing temperatures (Supplementary Figure S5). When the meconium lysis cocktails were frozen from 1 to 6 h, the efficiency was significantly improved, implying that the meconium lysis cocktails were fully lysed to release DNA and that the PCR inhibitors were inactive after freezing (Patton et al., 2007; Yadava et al., 2008). From 6 to 24 h, the extraction efficiency tended to be stable, while the efficiency slightly decreased when the samples were frozen for longer than 24 h. These results further proved that freezing treatments could improve DNA extraction efficiency. Regarding the time cost, 6 h was selected for the freezing time in the final protocol.

#### Different Thawing Operations

**Figure 2:**
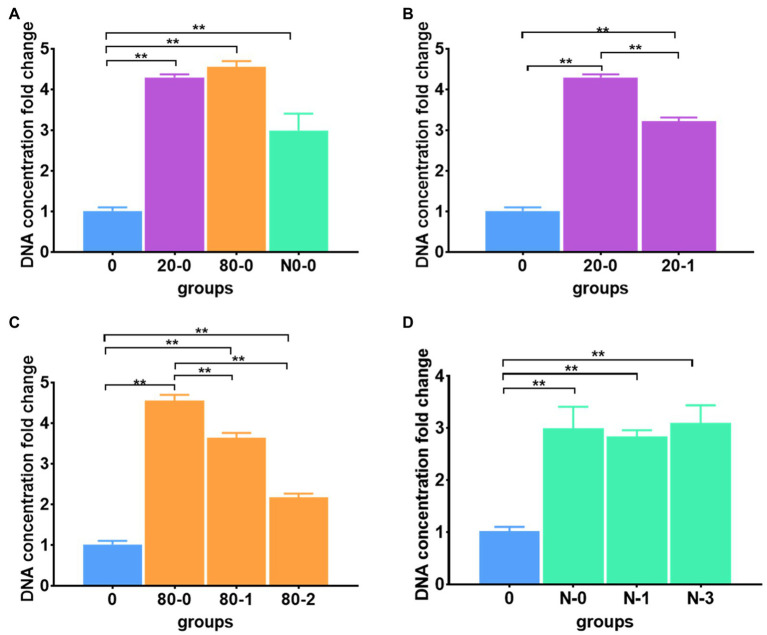
Different thawing protocols affected DNA extraction efficiency. **(A)** The DNA extraction efficiencies for immediately thawed meconium frozen at −20 and −80°C and in liquid nitrogen; **(B)** The DNA extraction efficiencies for immediately thawed and gradient thawed meconium frozen at −20°C; **(C)** The DNA extraction efficiencies for immediately thawed and gradient thawed meconium frozen at −80°C; **(D)** The DNA extraction efficiencies for immediately thawed and gradient thawed meconium frozen in liquid nitrogen. The corresponding data were shown in Supplementary Table S3. **P < 0.01.

Regarding thawing operations for the frozen meconium with the different protocols (immediate or gradient thawing) and for the different freezing patterns (freezing at −20°C or −80°C, or in liquid nitrogen), the DNA extraction efficiency changed significantly according to a comparison with the reference protocol (Figure 2; Supplementary Table S3). Among all immediately thawed meconium samples, the DNA extraction efficiencies for samples frozen at −20 and −80°C were much higher than those for samples frozen in liquid nitrogen; however, all freezing patterns can improve the efficiency compared to the reference (Figure 2A). These data suggested that freezing meconium at any investigated temperature with immediate thawing can enhance DNA extraction, and freezing at −20 and −80°C was more efficient than freezing in liquid nitrogen.

Combining the factor of immediate or gradient thawing, the DNA extraction efficiency of gradient thawing was significantly lower than that of immediate thawing for meconium frozen at −20°C (Figure 2B). Similarly, meconium frozen at −80°C had a similar trend to that frozen at −20°C, where the DNA yields with gradient thawing were obviously lower than those with immediate thawing (Figure 2C). However, for meconium frozen in liquid nitrogen, both immediate and gradient thawing had nearly the same efficiency for DNA extraction (Figure 2D).

The above results demonstrated that freezing pretreatments can improve DNA extraction efficiency. The immediate thaw operation has better extraction efficiency than gradient thawing. The liquid nitrogen freezing groups had significantly lower DNA extraction efficiencies than the other two freezing groups. We suspected that −20 and −80°C were the proper temperatures to facilitate DNA release and avoid DNA breaking in the meconium matrix. Therefore, the immediate thawing operation was selected for the final protocol.

#### Different Freezing Patterns

**Figure 3:**
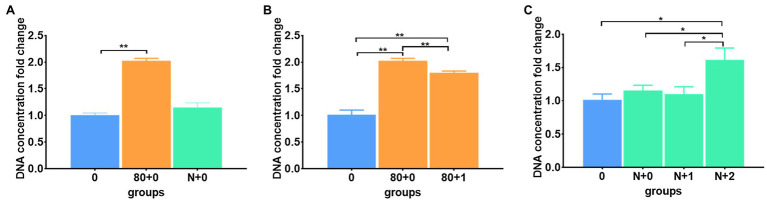
Different freezing patterns affected DNA extraction efficiency. **(A)** The DNA extraction efficiencies in the immediate freezing groups; **(B)** The DNA extraction efficiencies in the immediate and gradient freezing groups when samples were frozen at −80°C; **(C)** The DNA extraction efficiencies in the immediate and gradient freezing groups when samples were frozen in liquid nitrogen. The corresponding data were shown in Supplementary Table S4. *P < 0.05, **P < 0.01.

When comparing the different freezing temperatures (Figure 2A), both −20 and −80°C-frozen meconium samples showed significantly improved DNA yields after immediate thawing (Supplementary Table S4). In addition to the thawing operation, freezing by immediate and gradient cooling to −80°C and liquid nitrogen conditions were assessed (Figure 3A). The results showed that immediately cooling down to −80°C can result in a better improvement than gradient cooling (Figure 3B); however, immediate cooling in the liquid nitrogen condition showed no difference with gradient cooling in terms of the DNA extraction efficiency (Figure 3C), which implied that the temperature of freezing in liquid nitrogen was too low to damage the yield of DNA. Instead, the results suggested that freezing at −20 and −80°C was sufficient to improve DNA yields, and immediate cooling was better than gradient cooling.

#### Freeze-Thaw Duplication

**Figure 4:**
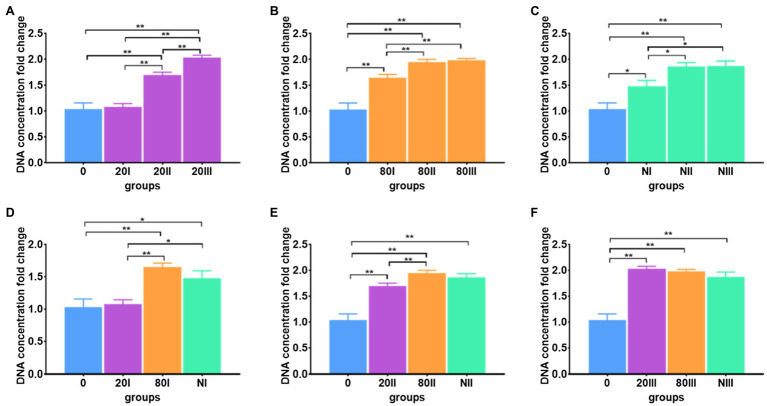
Freeze-thaw cycle number-dependent DNA extraction efficiency. **(A)** The freeze-thaw cycle-dependent DNA extraction efficiency for meconium frozen at −20°C; **(B)** The freeze-thaw cycle-dependent DNA extraction efficiency for meconium frozen at −80°C; **(C)** Freeze-thaw cycle-dependent DNA extraction efficiency for meconium frozen in liquid nitrogen; **(D)** The DNA extraction efficiencies with one freeze-thaw cycle; **(E)** The DNA extraction efficiencies with two freeze-thaw cycles; **(F)** The DNA extraction efficiencies with three freeze-thaw cycles. The corresponding data were shown in Supplementary Table S5. *P < 0.05, **P < 0.01.

The DNA extraction efficiencies of repeated freeze-thaw operations were investigated (Supplementary Table S5). For each freeze-thaw cycle, immediate cooling and thawing were carried out. For all freezing conditions of cooling to −20 and −80°C and freezing in liquid nitrogen, the DNA extraction efficiencies increased with freeze-thaw cycle number (Figures 4A–C). When comparing the freezing temperature-dependent efficiencies at each cycle (Figures 4D–F), the highest efficiency was achieved −20°C freeze-thaw operation after the 3rd cycle.

All data showed that freeze-thaw operation can improve the DNA extraction efficiency significantly, which may be due to the multiple freeze-thaw cycles that can result in the meconium lysis cocktails being completely lysed to release DNA. In addition to the total DNA extraction efficiency, bacterial DNA abundance in meconium and their stability during the investigated protocols are key to relating the final detection rate of PCR analysis (Patton et al., 2007; Yadava et al., 2008; Zhang et al., 2008; Chen et al., 2018).

### Bacterial DNA Abundance and Freezing Temperature Related Sequencing

**Figure 5:**
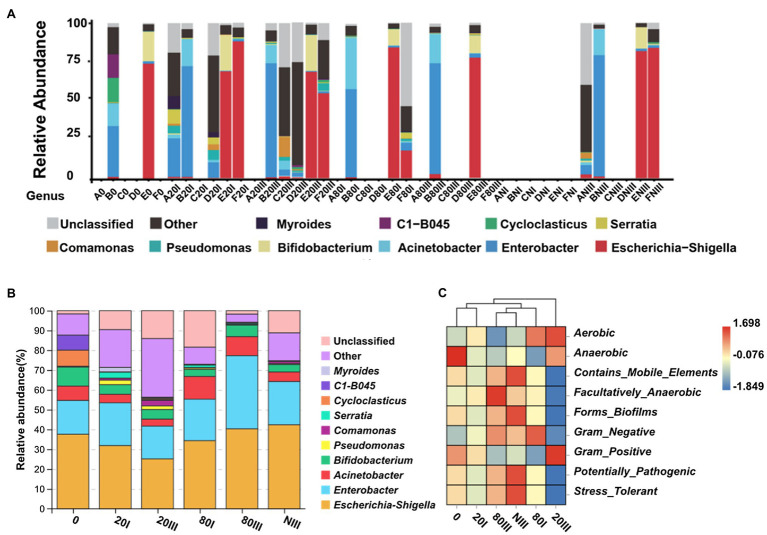
Relative abundance and functional prediction of bacteria at the genus level. **(A)** The relative abundance of bacterial DNA at the genus level from the perspective of sample. A0–F0: Two (B and E) of six samples could be positively sequenced for bacterial DNA by the reference protocol; A20I–F20I: Five (A, B, D, E, and F) of six samples could be positively sequenced for bacterial DNA with a one-cycle −20°C freeze-thaw protocol; A20III–F20III: Five (B, C, D, E, and F) of six samples could be positively sequenced for bacterial DNA with a three-cycle −20°C freeze-thaw protocol; A80I–F80I: Three (B, E, and F) of six samples could be positively sequenced for bacterial DNA with a one-cycle −80°C freeze-thaw protocol; A80III–F80III: Two (B and E) of six samples could be positively sequenced for bacterial DNA with a three-cycle −80°C freeze-thaw protocol; ANI–FNI: No samples could be positively sequenced for bacterial DNA with a one-cycle liquid nitrogen freeze-thaw protocol; ANIII–FNIII: Four (A, B, E, and F) of six samples could be positively sequenced for bacterial DNA with a three-cycle liquid nitrogen freeze-thaw protocol; **(B)** The relative abundance of bacterial DNA at the genus level from the perspective of group; **(C)** Functional prediction of bacterial DNA among five pretreated groups.

Six different meconium samples were sequenced for their 16S rDNA by reference DNA extraction (0) and six other protocols (20I: one freeze-thaw cycle at −20°C, 20III: three freeze-thaw cycles at −20°C, 80I: one freeze-thaw cycle at −80°C, 80III: three freeze-thaw cycles at −80°C, NI: one freeze-thaw cycle in liquid nitrogen, and NIII: three freeze-thaw cycles in liquid nitrogen) at different freezing temperatures, and freeze-thaw cycles were applied. The positive detection of meconium samples A, B, C, D, E, and F from high to low rates was 6 (B), 6 (E), 4 (F), 2 (D), 2 (A), and 1 (C). The PCR sequencing method has equal sensitivity to the different extraction protocol-treated samples. Meconium B and E can be sequenced positively by six protocols (Figure 5) except the NI protocol, which implied that B and E have the most abundant bacterial DNA and can meet 16S rDNA sequencing requirements for low or high DNA extraction efficiencies. However, meconium C can only be sequenced by protocol 20III (C20III), which has the highest DNA extraction efficiency (Figures 4A,F). Therefore, meconium C may have the lowest bacterial DNA abundance among the six samples. Meconium A can be sequenced by protocols 20I (A20I) and NIII, which may imply that bacterial DNA in A is slightly higher than that in C. Samples F and D may have bacterial DNA abundance levels lower than those in B and E because both D and F can be sequenced when treated by protocols 20I and 20III. In addition, meconium F can be sequenced by the protocol of 80I (F80I) and NIII (FNIII), which may imply that the level of bacterial DNA in F was slightly higher than that in D. In summary, the bacterial DNA levels from high to low in these samples were B ≈ E > F ≥ D > A ≈ C. At the median levels of bacteria, a −20°C freeze-thaw cycle could improve the DNA extraction efficiency.

Meconium can be sequenced not only because of its bacterial DNA abundance but also because of its DNA extraction efficiency and stability during the protocol. The positive detection of all meconium samples for the 0, 20I, 20III, 80I, 80III, NI, and NIII protocols from the high to low rates was 5 (20I), 5 (20III), 4 (NIII), 3 (80I), 2 (80III), 2 (0), and 0 (NI), respectively (Figure 5). Among all one cycle freeze-thaw protocols, the DNA extraction efficiencies of 80I and NI were higher than those of 20I (Figure 4D). However, five of six samples could be sequenced positively by the 20I protocol, and no sample could be sequenced by the NI protocol. These results suggested that too low of a freezing temperature may damage the bacterial DNA for sequencing. The DNA extraction efficiencies of all three freeze-thaw cycles were almost equal. However, the samples sequenced from high to low were 20III > NIII >80III. These results further supported that too low of a freezing temperature may damage bacterial DNA sequencing. When comparing their DNA extraction efficiencies (Figures 4A–D), three freeze-thaw cycles were higher than one freeze-thaw cycle. However, five of six samples could be sequenced positively by both 20I and 20III protocols, which implied that 20I and 20III were the most effective protocols for sample sequencing. The meconium C with the lowest bacterial DNA abundance could only be sequenced by pretreatment with the 20III protocol, which implied that 20III was more effective than 20I. Regardless, illustrating that meconium A can be sequenced by 20I and NIII but not by 20III is difficult. In summary, the protocol with −20°C pretreatment showed the highest rates of bacterial DNA sequencing, and the reference and liquid nitrogen pretreatment protocols showed the lowest rates. Therefore, −20°C was the best pretreatment temperature.

The microbial species diversity at the genus level was different according to the different DNA extraction protocols. The number of genera was calculated on the fully profiled bacterial DNA in meconium A, B, C, D, E, and F (Figure 5A), which is listed in Table 1 and visualized in Supplementary Figures S6A–D. All results indicated that pretreatment at −20°C yielded the highest genera, which was additional evidence that −20°C was the best choice, possibly because −20°C has more influence on DNA integration than −80°C, and temperatures that are too low could break some bacterial DNA with high genera diversity (Supplementary Figures S6A–E); however, the destructiveness of organic tissue at −3 to −10°C (Khan and Vincent, 1996; Bortolin et al., 2017) may have been reduced. When comparing the genera numbers between the 20I and 20III pretreatment groups, the 20III was higher than 20I in samples B, D, and F but the 20I was higher than 20III in samples E. Generally, pretreatment of three freeze-thaw cycles at −20°C can retain the highest DNA genera diversity, and this may be the optimal protocol to extract meconium. Analyses of α diversity further confirmed the above conclusion, which showed 20I generated the highest DNA genera diversity, followed by 20III, while α diversity was almost equal between 80I and 80III (Supplementary Figure S8). Species analysis presented that 20III dominated by other genera (29.64%), *Escherichia-Shigella* (25.16%), and *Enterobacter* (16.53%), while control group was dominated by *Escherichia-Shigella* (37.56%), *Enterobacter* (17.07%), and other genera (10.76%) (Figure 5B). These results partially confirmed the conclusions conducted by Stinson et al. (2019), and the relatively high abundance of other genera in 20III group indicated freeze-thaw pretreatment would be beneficial to the release of low abundance bacterial DNA. And by utilizing this protocol, the relative abundances of genera of *Akkermansia* (*p* = 0.047), *Ruminococcus* (*p* = 0.010), *Acidipila* (*p* = 0.011), and *Caulobacter* (*p* < 0.001) were significantly higher comparing with the 20I group, while no genus was detected to be decreased in our study according to Wilcoxon analysis. In addition, the major effects of 20III pretreatment was shown to be associated with the increased diversity of aerobic, anaerobic, and Gram-positive bacteria, which was hypothesized that the freeze-thaw method mainly promoted the release of bacterial DNA with such characteristics (Figure 5C).

**Table 1:** The genus numbers measured in each group.

Sample/protocol	0	20I	20III	80I	80III	NI	NIII
A	/	196	/	/	/	/	117
B	131	145	188	122	95	/	93
C	/	/	237	/	/	/	/
D	/	139	172	/	/	/	/
E	80	195	188	128	131	/	98
F	/	188	201	156	/	/	125

Meconium samples and the related DNA extraction protocols are marked by using the same codes as in Figure 5.

However, several limitations of this research are noted. One is that DNA-based assessments of low microbial biomass samples are extremely prone to confounding findings from contaminant DNA. Despite the rigorous sterile operations were adopted, bacterial DNA from reagents, consumables, and components of DNA extraction kits may not be avoided, which may affect the reliability of our results (Tanner et al., 1998; Grahn et al., 2003; Mühl et al., 2010). The other limitation is that our conclusions were largely based on the macroscopic descriptions. The specific mechanisms for enhancing the DNA extraction efficiency by freeze-thaw pretreatment were unexplored, as well as rare in the previous researches. Therefore, explaining these mechanisms was urgently demanded, and that could be one of the future research directions.

## Conclusion

Microbial DNA in meconium was difficult to extract using general commercial stool DNA extract kits due to the extremely low microbial biomass. The amplification rate of extracted DNA from meconium is usually approximately 10–52.9% (Ardissone et al., 2014; Hansen et al., 2015). In this assay, we found that freeze-thaw recycling pretreatment can enhance the efficiency of bacterial DNA extraction from meconium, and an improved protocol was developed and verified using the commercial QIAamp® Fast DNA Stool Mini Kit (cat. no. 51604).

Finally, we concluded that (1) 30 mg lyophilized meconium was the optimal amount for DNA extraction. (2) Lysis cocktail freezing can improve DNA extraction efficiency, with 6 h being a suitable freezing time. (3) To improve DNA extraction efficiency, thawing the frozen lysis cocktail immediately is better than thawing gradually; similarly, freezing the lysis cocktail immediately is better than freezing gradually. (4) Regarding freezing temperatures, −20°C better maintains the stability of bacterial DNA than at −80°C and liquid nitrogen. (5) Finally, we suggested that three cycles of freeze-thaw treatment of the meconium lysis cocktail at −20°C can markedly increase the bacterial DNA extraction amount and genus diversity. In summary, this work offers a protocol to enhance meconium bacterial DNA extraction, which may be helpful to promote the study of foetal and/or newborn microbiome exposure.

## Data Availability Statement

The raw data of 16S rRNA sequence used in this study have been deposited at NCBI database under the accession number of PRJNA759695.

## Ethics Statement

The studies involving human participants were reviewed and approved by the Ethics Committee of the Xiamen Maternity and Child Health Care Hospital (XMCH), Xiamen, China. Written informed consent to participate in this study was provided by the participants’ legal guardian/next of kin.

## Author Contributions

YXin, JX, BN, CT, QW, YXia, and YL designed and performed the experiments and analyzed the data. YXin wrote the original manuscript. HS and XZ designed the study and edited the manuscript. All authors contributed to the article and approved the submitted version.

## Funding

This work was financially supported by the Xiamen Medical and Health Project (Instructional Project; No. 3502Z20189050), Xiamen Medical and Health Key Project (No. 3502Z20191102), and Xiamen Science and Technology Plan Project (No. 3502Z20199138).

## Conflict of Interest

The authors declare that the research was conducted in the absence of any commercial or financial relationships that could be construed as a potential conflict of interest.

## Publisher’s Note

All claims expressed in this article are solely those of the authors and do not necessarily represent those of their affiliated organizations, or those of the publisher, the editors and the reviewers. Any product that may be evaluated in this article, or claim that may be made by its manufacturer, is not guaranteed or endorsed by the publisher.

## Footnotes

^1^ http://www.omicsmart.com
