# Supplementary material for: Freeze-Thaw Pretreatment Can Improve Efficiency of Bacterial DNA Extraction From Meconium
Source: Front Microbiol. 2021 Dec 9;12:753688. doi: 10.3389/fmicb.2021.753688 (PMC8695897; doi:10.3389/fmicb.2021.753688)
Supplement: Supplementary file 1 [file Data_Sheet_1.pdf]

Supplementary Materials

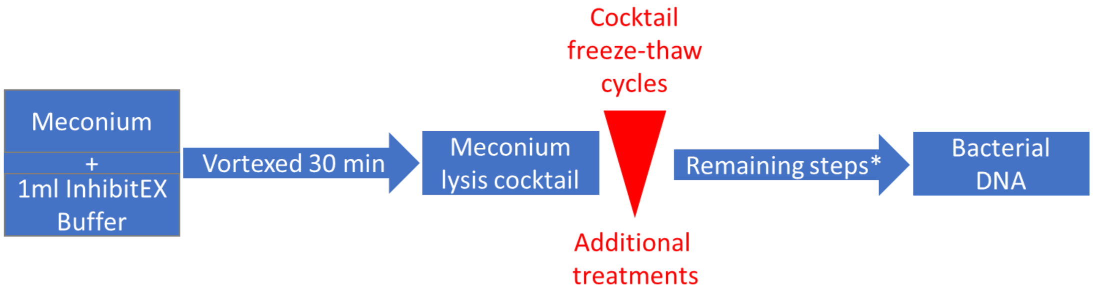

Figure S1. Basic protocol and additional treatments for bacterial DNA extraction.

Note: Each sample was mixed with 1 ml InhibitEX buffer and vortexed for 30 min. Then, the meconium lysis cocktail was treated by the remaining steps of the DNA extraction protocol (blue blocks). In the present study, freeze-thaw cycles (red block) were added to enhance DNA release into the cocktail. *The remaining steps are the operational steps offered by the production instructions of the kit.

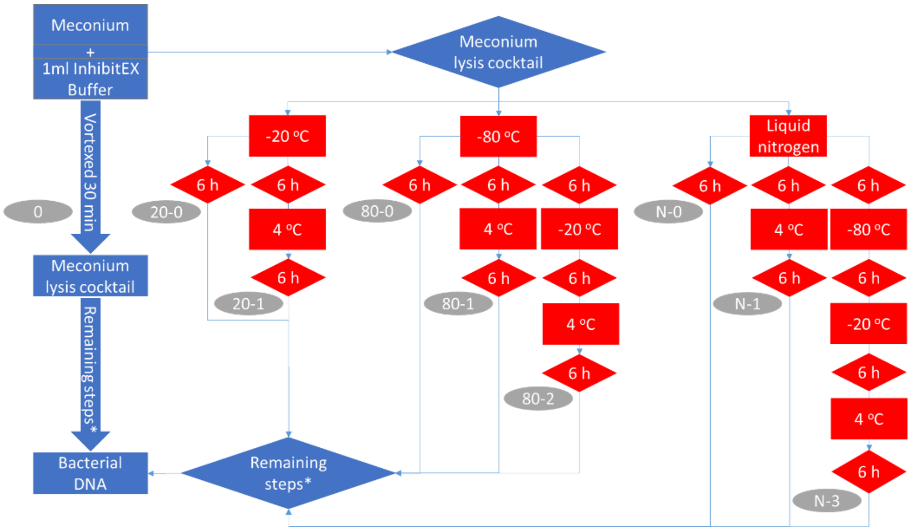

Figure S2. Tests of immediate and gradient thawing steps.

0: Reference of the samples treated by following the remaining basic steps after vortex; 20-0: Meconium lysis cocktails frozen at -20 ℃ for 6 h and thawed at room temperature immediately for DNA extraction by following the remaining basic steps; 20-1: Meconium lysis cocktails frozen at -20 ℃ for 6 h, thawed at 4℃ for 6 h and at room temperature for DNA extraction by following the remaining basic steps; 80-0: Meconium lysis cocktails frozen at -80 ℃ for 6 h and thawed at room temperature immediately for DNA extraction by following the remaining basic steps; 80-1: Meconium lysis cocktails frozen at -80 ℃ for 6 h, thawed at 4 ℃ for 6h and at room temperature for DNA extraction by following the remaining basic steps; 80-2: Meconium lysis cocktails frozen at -80 ℃ for 6 h, thawed at -20 ℃ for 6 h, 4 ℃ for 6h, and at room temperature for DNA extraction by following the remaining basic steps; N-0: Meconium lysis cocktails frozen in liquid nitrogen for 6 h and thawed at room temperature immediately for DNA extraction by following the remaining basic steps; N-1: Meconium lysis cocktails frozen in liquid nitrogen for 6 h, thawed at 4 ℃ for 6h and at room temperature for DNA extraction by following the remaining basic steps; N-3: Meconium lysis cocktails frozen in liquid nitrogen for 6 h, thawed at -80 ℃ for 6 h, -20 ℃ for 6h, 4℃ for 6h, and at room temperature for DNA extraction by following the remaining basic steps.

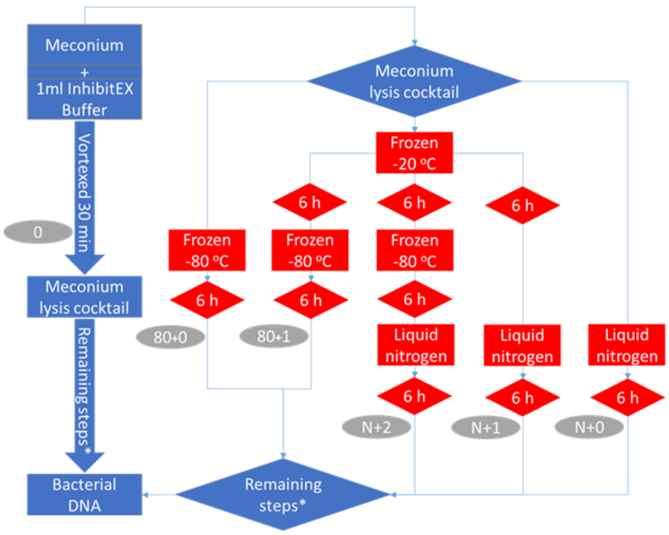

Figure S3. Tests of immediate and gradient freezing measures.

0: Reference of the samples treated by following the remaining basic steps after vortex; 80+0: Meconium lysis cocktails immediately frozen at -80 ℃ for 6 h and immediately thawed at room temperature for DNA extraction by following the remaining basic steps; 80+1: Meconium lysis cocktails immediately frozen at -20 ℃ for 6 h and then transferred to -80 ℃ for 6 h; finally, the cocktails immediately thawed at room temperature for DNA extraction by following the remaining basic steps; N+0: Meconium lysis cocktails immediately frozen in liquid nitrogen for 6 h and immediately thawed at room temperature for DNA extraction by following the remaining basic steps; N+1: Meconium lysis cocktails immediately frozen at -20 ℃ for 6 h and then transferred into liquid nitrogen for 6 h; finally, the cocktails immediately thawed at room temperature for DNA extraction by following the remaining basic steps; N+2: Meconium lysis cocktails immediately frozen at -20 ℃ for 6 h and then transferred to -80 ℃ for 6 h and in liquid nitrogen for 6 h, respectively; finally, the cocktails immediately thawed at room temperature for DNA extraction by following the remaining basic steps.

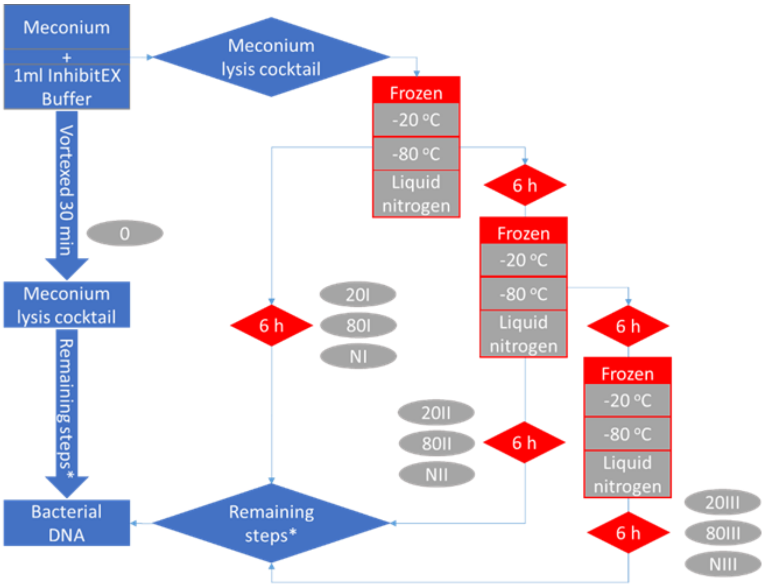

Figure S4. Tests of freeze-thaw recycling times.

0: Reference of the samples treated by following the remaining basic steps after vortex; 20I, 80I and NI: After the 1st cycle freeze-thaw, the meconium lysis cocktails were immediately frozen at -20 ℃ (20I), -80 ℃ (80I) and in liquid nitrogen (NI) for 6 h, respectively; then the cocktails were thawed immediately at room temperature for DNA extraction by following the remaining basic steps; 20II, 80II and NII: After the 2nd cycle of freeze-thaw, the meconium lysis cocktails were immediately frozen at -20 ℃ (20II), -80 ℃ (80II) and in liquid nitrogen (NII) for 6 h, respectively; then the cocktails were thawed immediately at room temperature for DNA extraction by following the remaining basic steps; 20III, 80III and NIII: After the 3rd cycle freeze-thaw, the meconium lysis cocktails were immediately frozen at -20 ℃ (20III), -80 ℃ (80III) and in liquid nitrogen (NIII) for 6 h, respectively; then the cocktails were thawed immediately at room temperature for DNA extraction by following the remaining basic steps.

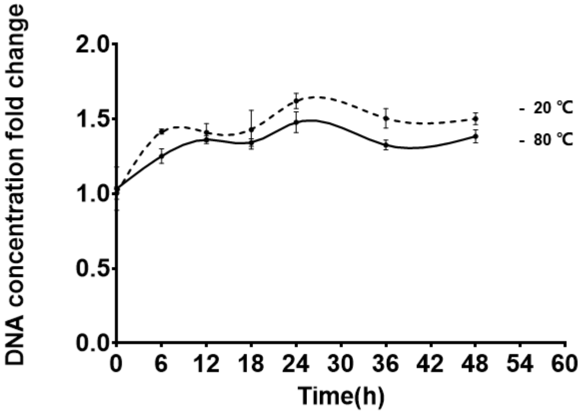

Figure S5. Trends of freezing time period related DNA concentration fold change.

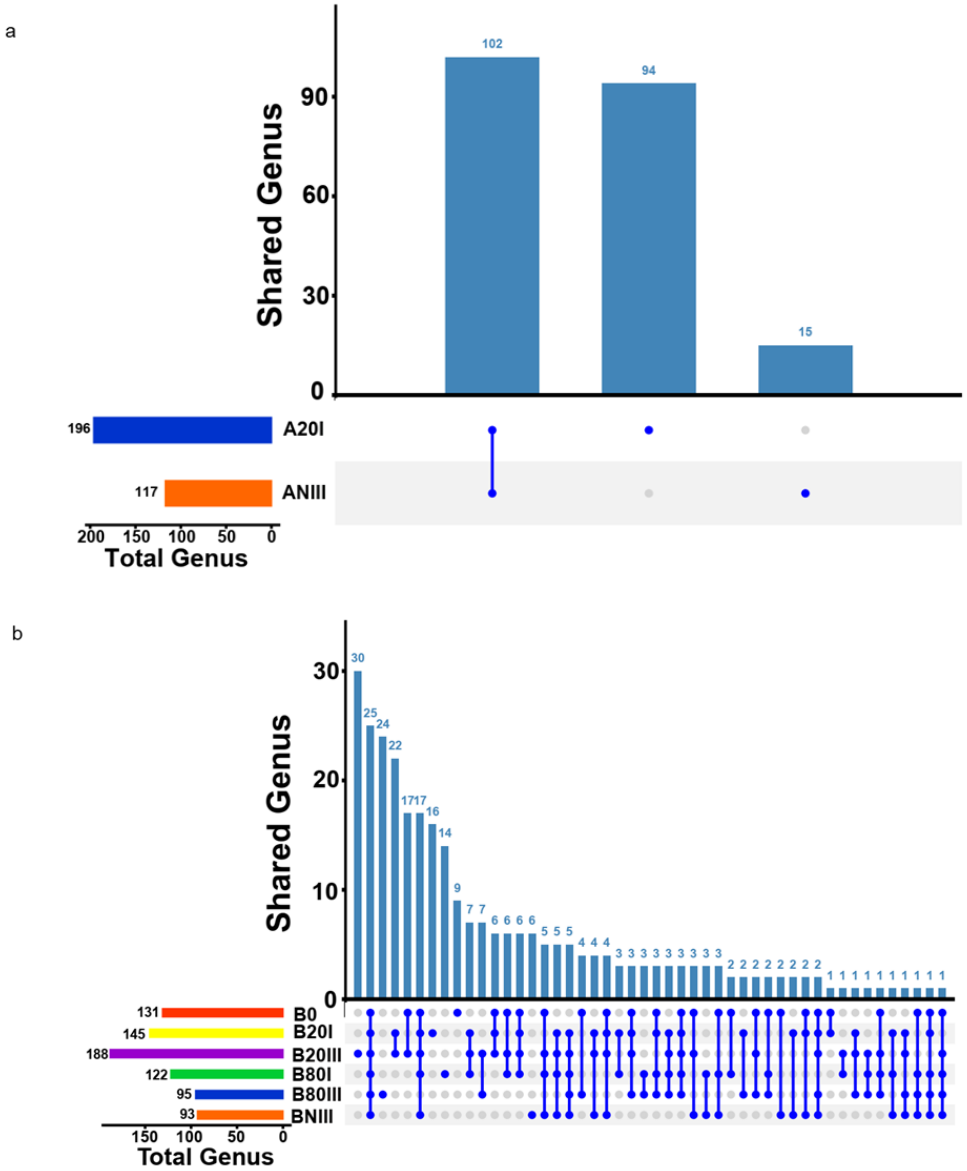
a
Shared Genus
102
94
15
196
A20I
117
ANIII
Total Genus
b
Shared Genus
131
B0
145
B20I
188
B20III
122
B80I
95
B80III
93
BNIII
Total Genus

c

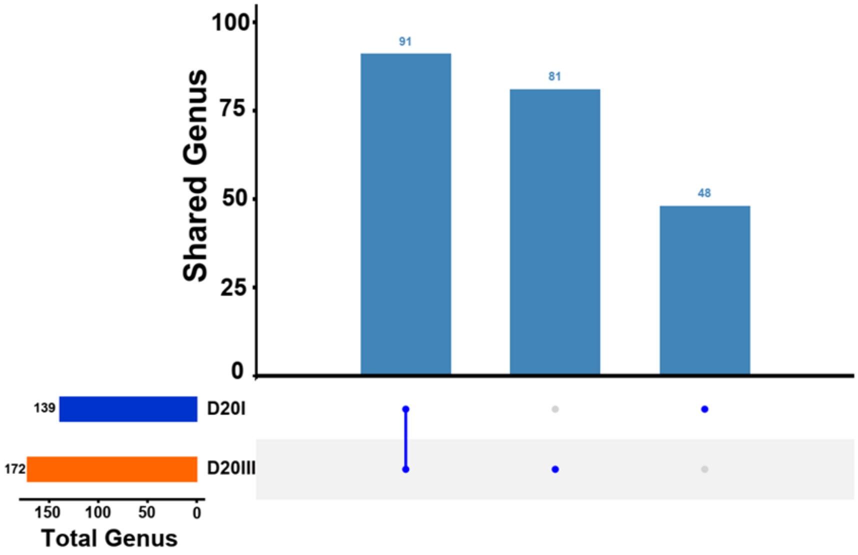
Shared Genus
100
75
50
25
0
91
81
48
139
D20I
172
D20III
150 100 50 0
Total Genus

d

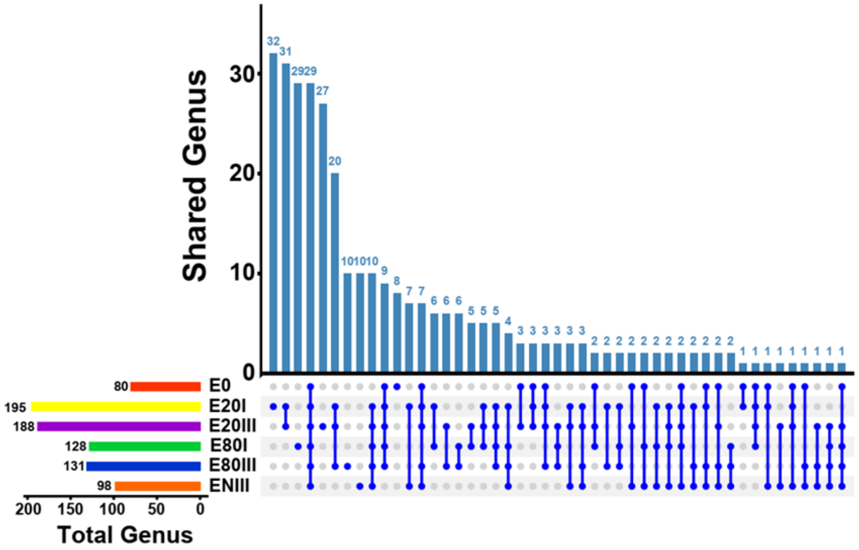
Shared Genus
30
20
10
0
32 31 29 29 27 20 10 10 10 9 8 7 7 6 6 6 5 5 5 4 3 3 3 3 3 3 2 2 2 2 2 2 2 2 2 2 2 2 1 1 1 1 1 1 1 1 1
80 E0
195 E20I
188 E20III
128 E80I
131 E80III
98 ENIII
200 150 100 50 0
Total Genus

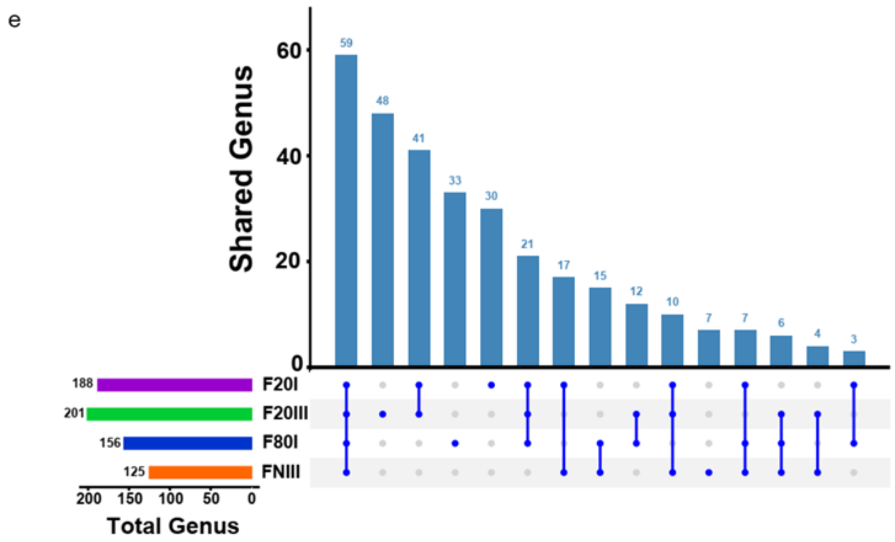

Figure S6. Genera number variation of the detected meconium samples.

a. The upset plot of group A; b. The upset plot of group B; c. The upset plot of group D; d. The upset plot of group E; e. The upset plot of group F. Meconium samples and the related DNA extraction protocols have been marked by using the same codes as in Figure 5.

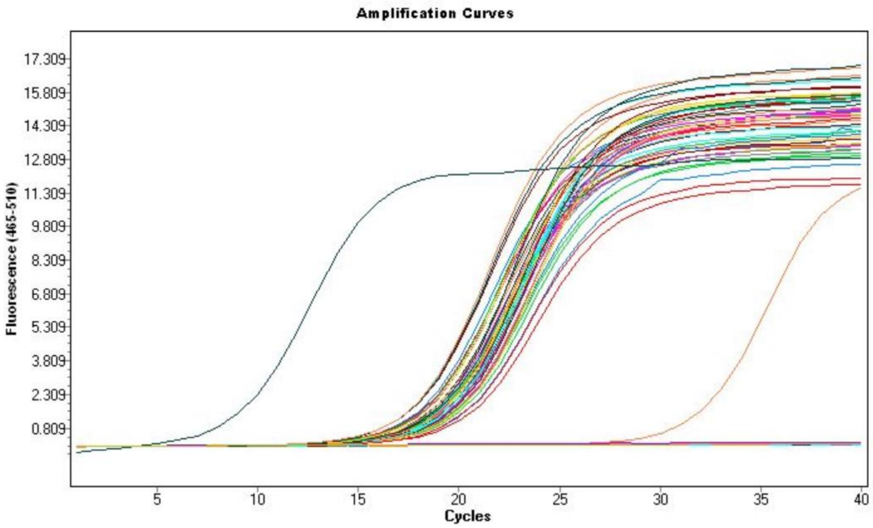

Figure S7. Amplification curves of the detected meconium samples.

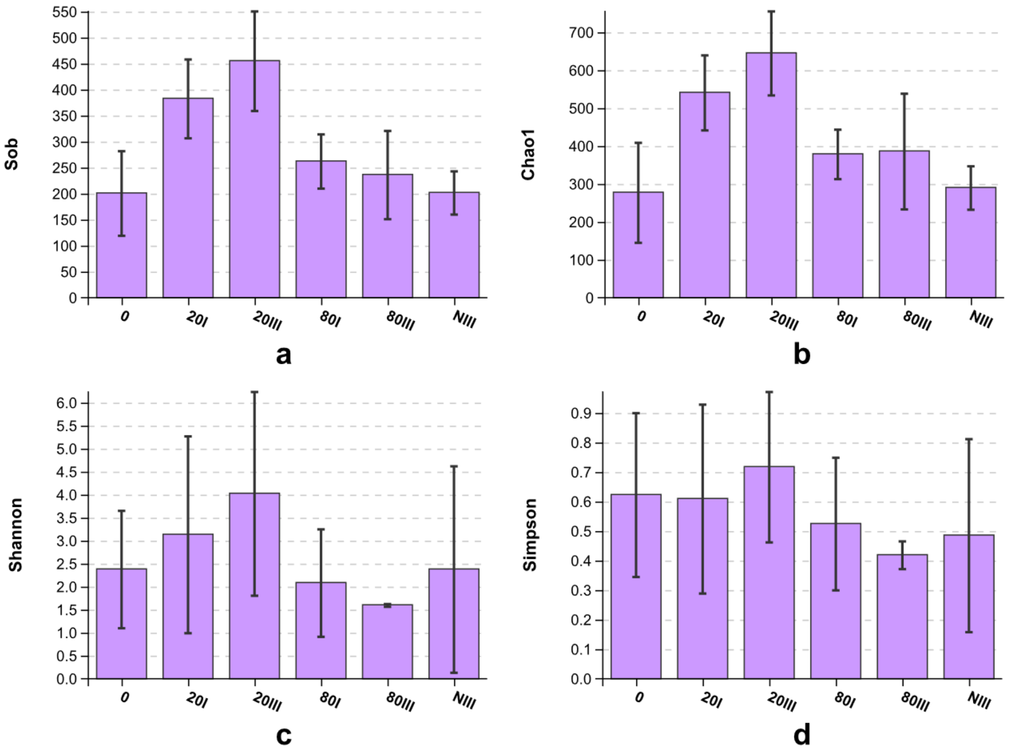

Figure S8. Amplification curves of the detected meconium samples.

Table S1. Water content of each sample

**Loading amount of meconium sample**					
**Fresh (mg)**	**40**	**80**	**120**	**160**	**200**
Water content (%)	55.20 ± 6.96	55.16 ± 8.96	55.46 ± 4.09	56.79 ± 5.77	54.92 ± 12.01

Table S2. Effects of meconium status and loading amount on DNA extraction efficiency

Meconium loading amount related DNA extraction						
**Fresh (mg)**	**40**	**80**	**120**	**160**	**200**	
CT values	21.96 ± 1.39	18.44 ± 0.55	16.37 ± 2.21	16.94 ± 0.24	17.80 ± 0.47	
DNA concentrations (ng/μL)	31.32 ± 2.36	50.60 ± 2.66	70.68 ± 2.44	62.74 ± 1.76	43.82 ± 3.23	
**Freeze-dried (mg)**	**10**	**20**	**30**	**40**	**50**	**60**
CT values	21.09 ± 0.18	19.80 ± 0.60	19.45 ± 0.43	20.68 ± 0.13	22.53 ± 0.25	22.41 ± 0.35
DNA concentrations (ng/μL)	36.06 ± 3.25	43.88 ± 3.28	48.08 ± 2.61	40.58 ± 3.36	29.82 ± 2.89	30.42 ± 3.97

CT values and DNA concentrations after qPCR for 45 cycles of fresh and freeze-dried meconium samples are presented in the table. The results are shown as the average of 5 duplicates ± Standard Error of Mean (SEM).

Table S3. Different thawing protocols affected DNA extraction efficiency

Immediately and gradually thawing protocols									
Protocols	**0**	**20-0**	**20-1**	**80-0**	**80-1**	**80-2**	**N-0**	**N-1**	**N-3**
CT values	19.22 ± 0.41	16.12 ± 0.10	16.74 ± 0.14	16.00 ± 0.15	16.48 ± 0.14	17.57 ± 0.19	17.00 ± 0.80	17.01 ± 0.19	16.87 ± 0.51
P	-	<0.001	<0.001	<0.001	<0.001	<0.001	<0.001	<0.001	<0.001
DNA concentrations (ng/μL)	46.26 ± 3.76	66.66 ± 3.11	62.58 ± 3.47	69.48 ± 2.38	62.84 ± 1.91	43.22 ± 12.88	59.30 ± 6.45	46.38 ± 8.00	51.02 ± 12.46
P	-	<0.001	<0.001	<0.001	<0.001	0.626	0.005	0.977	0.437

The DNA extraction efficiencies (CT values and DNA concentrations after qPCR for 45 cycles) for immediately thawing protocols (i.e., 20-0 for -20 °C and 80-0 for -80 °C and N-0 for liquid nitrogen) and gradually thawing protocols (i.e., 20-1 for -20 °C and 80-1 and 80-2 for -80 °C and N-1, N-2 and N-3 for liquid nitrogen) of frozen meconium. The results are shown as the average of 5 duplicates ± Standard Error of Mean (SEM). Each group was statistically compared with Group "0" (control group). *P* was calculated using two-tail Student's t test with 0.05 as the threshold.

Table S4. Different freezing protocols affected DNA extraction efficiency

	Immediately and gradually freezing protocols					
**Protocol**	**0**	**80+0**	**80+1**	**N+0**	**N+1**	**N+2**
CT values	18.36 ± 0.20	16.87 ± 0.11	17.12 ± 0.17	18.09 ± 0.33	18.21 ± 0.46	17.40 ± 0.45
P	-	<0.001	<0.001	0.007	0.140	<0.001
DNA concentrations (ng/μL)	46.52 ± 2.33	54.54 ± 2.83	53.42 ± 2.83	47.66 ± 6.23	47.02 ± 4.32	49.60 ± 6.64
P	-	0.007	0.030	0.703	0.747	0.190

The DNA extraction efficiencies (CT values and DNA concentrations after qPCR for 45 cycles) for immediately freezing protocols (i.e., 80+0 for -80 °C and N+0 for liquid nitrogen) and gradually freezing protocols (i.e., 80+1 for -80 °C and N+1, N+2 for liquid nitrogen) of frozen meconium. The results are shown as the average of 5 duplicates ± Standard Error of Mean (SEM). Each group was statistically compared with Group "0" (control group). *P* was calculated using two-tail Student's t test with 0.05 as the threshold. Only one freezing protocol was designed for -20°C freezing.

Table S5. Freeze-thaw cycle number-dependent DNA extraction efficiency

Freeze-thaw cycle related DNA extraction										
**Protocol**	**0**	**20I**	**20II**	**20III**	**80I**	**80II**	**80III**	**NI**	**NII**	**NIII**
CT values	19.37 ± 0.59	19.23 ± 0.30	18.25 ± 0.15	17.87 ± 0.12	18.31 ± 0.17	17.96 ± 0.14	17.92 ± 0.09	18.56 ± 0.33	18.06 ± 0.18	18.04 ± 0.23
P	-	0.559	<0.001	<0.001	<0.001	<0.001	<0.001	0.016	<0.001	<0.001
DNA concentrations (ng/μL)	43.34 ± 4.00	46.22 ± 2.44	50.14 ± 2.91	57.04 ± 2.30	49.22 ± 1.70	51.70 ± 4.15	53.20 ± 3.26	48.08 ± 3.93	50.88 ± 3.26	50.64 ± 3.48
P	-	0.207	0.015	<0.001	0.016	0.012	0.03	0.096	0.011	0.015

The freeze-thaw cycle-dependent DNA extraction efficiencies (CT values and DNA concentrations after qPCR for 45 cycles) for meconium frozen at -20 °C (20I, 20II and 20III), -80 °C (80I, 80II and 80III), and in liquid nitrogen (NI, NII and NIII). The numbers of freeze-thaw cycles are indicated by I, II and III, respectively. Group "0" was served as control group for statistical comparison. The results are shown as the average of 5 duplicates ± Standard Error of Mean (SEM). *P* was calculated using two-tail Student's t test with 0.05 as the threshold.
